# Inflammatory Patterns Associated with *Legionella* in HIV and Pneumonia Coinfections

**DOI:** 10.3390/pathogens13020173

**Published:** 2024-02-14

**Authors:** Breanne M. Head, Adriana Trajtman, Ruochen Mao, Kathryn Bernard, Lázaro Vélez, Diana Marin, Lucelly López, Zulma Vanessa Rueda, Yoav Keynan

**Affiliations:** 1 Department of Medical Microbiology and Infectious Diseases, University of Manitoba, Winnipeg, MB R3E 0J9, Canada; breanne.head@phac-aspc.gc.ca (B.M.H.); maotony@hotmail.com (R.M.); zulma.rueda@umanitoba.ca (Z.V.R.); 2 National Microbiology Laboratory, Public Health Agency of Canada, Winnipeg, MB R3E 3P6, Canada; kathy.bernard@phac-aspc.gc.ca; 3 School of Medicine, Universidad de Antioquia, Medellin 050010, Colombia; lazaro.velez@udea.edu.co; 4 Infectious Diseases Section, Hospital Universitario San Vicente Fundación, Medellin 050010, Colombia; 5 School of Medicine, Universidad Pontificia Bolivariana, Medellin 050010, Colombia; dianamarcela.marin@upb.edu.co (D.M.); lucelly.lopez@upb.edu.co (L.L.); 6 Department of Internal Medicine, University of Manitoba, Winnipeg, MB R3A 1R9, Canada; 7 Department of Community Health Sciences, University of Manitoba, Winnipeg, MB R3E 0W3, Canada

**Keywords:** bronchoalveolar lavage, cytokines, HIV, *Legionella*, pneumonia

## Abstract

*Legionella* infections have a propensity for occurring in HIV-infected individuals, with immunosuppressed individuals tending to present with more severe disease. However, understanding regarding the *Legionella* host response in immune compromised individuals is lacking. This study investigated the inflammatory profiles associated with *Legionella* infection in patients hospitalized with HIV and pneumonia in Medellín, Colombia from February 2007 to April 2014, and correlated these profiles with clinical outcomes. Sample aliquots from the Colombian cohort were shipped to Canada where *Legionella* infections and systemic cytokine profiles were determined using real-time PCR and bead-based technology, respectively. To determine the effect of *Legionella* coinfection on clinical outcome, a patient database was consulted, comparing laboratory results and outcomes between *Legionella*-positive and -negative individuals. Principal component analysis revealed higher plasma concentrations of eotaxin, IP-10 and MCP-1 (*p* = 0.0046) during *Legionella* infection. Individuals with this immune profile also had higher rates of intensive care unit admissions (adjusted relative risk 1.047 [95% confidence interval 1.027–1.066]). Results demonstrate that systemic markers of monocyte/macrophage activation and differentiation (eotaxin, MCP-1, and IP-10) are associated with *Legionella* infection and worse patient outcomes. Further investigations are warranted to determine how this cytokine profile may play a role in *Legionella* pneumonia pathogenesis or immunity.

## 1. Introduction

*Legionella*, and in particular *L. pneumophila* serogroup 1, are increasingly recognized as a major cause of pneumonia, accounting for $434 million in hospitalization claims annually [1,2]. *Legionella* pneumonia often presents as nonspecific symptoms (cough, fever, malaise, dyspnea, etc.) [3], accompanied by pulmonary infiltrates, pleural effusions, and radiographic deterioration [3,4]. Swift and accurate diagnosis and treatment are crucial to prevent significant morbidity and mortality. In cases of severe infection, individuals with *Legionella* pneumonia frequently need hospitalization [4], particularly when complications arise such as acute lung injury, hypoxemia, and pulmonary fibrosis, necessitating admission to the intensive care unit (ICU) [5,6].

Amidst the rising trends in *Legionella* infections [7,8,9], knowledge of the host response to these infections is not well understood. During infection, *Legionella* infiltrates pulmonary macrophages and evades the phagosomal maturation process by establishing a specialized replication structure known as the *Legionella* containing vacuole [10]. As the bacterium multiplies, its Dot/Icm type IV secretion system discharges effector proteins into the host cytosol, manipulating the cell to favor its intercellular growth and survival [11]. Despite playing a role in the progression of *Legionella* infections, macrophages also assume a pivotal function in instigating the host inflammatory response [12,13]. Upon bacterial detection, resident macrophages prompt the release of inflammatory mediators from epithelial cells which, in turn, leads to the recruitment of neutrophil and monocyte cells, triggering a Th1-predominant response and eventual cell death [14], albeit understanding of the precise mechanisms and cytokines involved are still incomplete.

In response to *Legionella* infection, there is an upregulation of the macrophage mitogen-activated protein kinase signaling pathway [15,16], a process crucial for regulating the immune response and producing immunomodulatory cytokines such as tumor necrosis factor (TNF)-α, interleukin (IL)-1, IL-10, and IL-12 [17]. Other cytokines thought to contribute to the defense against *Legionella* infection also include interferon (IFN)-γ, IL-2, IL-4, IL-6, IL-8, IL-12, IL-18, and monocyte chemoattractant protein 1 (MCP-1) [13,15,18].

In populations with compromised immune systems, such as those with chronic lung disease, or those on TNF-α inhibitors or immunomodulatory drugs, higher rates of *Legionella* infection have been reported [13,18]. Recent studies also highlight a propensity for *Legionella* infections to occur in people living with HIV [19,20]. Moreover, immunosuppressed HIV-infected individuals have been shown to present with a more severe course of disease, resulting in higher mortality rates [20,21]. Despite these findings, there is a notable lack of studies investigating the role that *Legionella* infections play in the causation and consequences of pneumonia among people living with HIV. Additionally, an understanding regarding the host cytokine secretion during *Legionella* spp. in HIV-infected individuals is lacking. Thus, the aim of this study was to investigate the inflammatory profile associated with *Legionella* spp. infections in HIV and pneumonia coinfected individuals and to correlate these profiles with clinical outcomes.

## 2. Materials and Methods

### 2.1. Population, Samples, and Ethics

This investigation utilized bronchoalveolar lavage (BAL) and plasma samples obtained from a cohort of individuals with HIV and pneumonia. The cohort consisted of patients admitted to Hospital Universitario San Vicente Fundación in Medellín, Colombia, between February 2007 and April 2014 [22,23]. Inclusion criteria for the original cohort included adults aged 18 years and older, diagnosed with both HIV and pneumonia, and those that underwent bronchoscopy with BAL sample collection [24] at the time of hospital admission. Exclusion criteria involved individuals with underlying lung conditions other than pneumonia, hospitalization within the last 14 days, recent antibiotic exposure (within 3 days of admission), or any other non-HIV-related immunodeficiencies. Informed consent was obtained from all study participants and ethics approvals were received from both the University of Manitoba (Winnipeg, Canada) and the Universidad de Antioquia (Medellín, Colombia).

### 2.2. In-Hospital Procedures

In hospital, BAL samples underwent microbiological investigations (culture and staining) based on findings from the clinical assessment. For culture, samples were plated on standard agar for bacterial quantification [25]. Additionally, samples were plated on Mycosel, Girasol, and Sabouraud’s agar for fungal assessment, and on OgawaKudoh medium and thin-layer agar [26] for mycobacteria cultivation. Stains of interest included Wright, Gram, Ziehl–Neelsen, modified Kinyoun, and modified toluidine blue, according to the Kahn–Jones’ protocol [27].

During care, patients were admitted to the ICU based on patient monitoring, need for mechanical ventilation, and hospital recommendation, and details were recorded in patient clinical charts. Patient data, including past medical history, symptoms, physical findings, laboratory and microbiology results, and outcomes (ICU admission, intubation, mechanical ventilation, and mortality), were collected from hospital clinical charts and collated into a patient database.

Aliquots of BAL and plasma samples were shipped to Winnipeg, Canada for further analysis.

### 2.3. Legionella Identification and Immunological Array Assay

*Legionella* identification and cytokine analysis were determined retrospectively and independently. Researchers were blinded to results as well as the patient database until all sample assays were completed.

To identify *Legionella* infections, bacterial DNA was extracted from patient BAL samples and *Legionella*-positive controls using the QIAamp DNA Microbiome Kit (QIAGEN, Hilden, Germany) following the manufacturer’s instructions. The DNA was aliquoted and stored at −20 °C. For *Legionella* qPCR, primer and probe sequences and assay conditions were adapted from Benitez et al. [28] and Cross et al. [29]. Primer and probe oligonucleotides (Integrated DNA Technologies, Coralville, IA, USA) were aliquoted and stored at −20 °C until required.

BAL DNA and positive control DNA were screened for *Legionella* by targeting a section of the 23 S–5 S intergenic spacer region, which encompasses both conserved and variable regions. Each PCR reaction contained the following: 12.5 μL of (2X) PrimeTime Gene Expression Mastermix (Integrated DNA Technologies, Coralville, IA, USA), 5 μL of template, 150 nM of each primer, and 100 nM of probe. All reactions were run using a C1000 Touch Thermal Cycler (Bio-Rad, Mississauga, ON, Canada). Cycling conditions consisted of an initial denaturation step of 95 °C for 300 s then 45 PCR cycles of 15 s at 95 °C and 60 s at 60 °C. Following qPCR, data analysis was performed using the CFX Manager System Software, Version 3.1 (Bio-Rad, Mississauga, ON, Canada). Samples were considered positive if they amplified with a sigmoidal curve and had a crossing threshold value greater than 10. All samples were run in duplicate and any discordant results were resolved using a third replicate.

Immunological testing was performed on plasma, cell-free supernatants using a Bio-Plex Pro Human Cytokine Magnetic 19-Plex Panel (Bio-Rad, Mississauga, ON, Canada) on the Bio-Plex platform (version 6.0 software) and was carried out according to the manufacturers’ instructions. Targets of interest included: IFN-γ, TNF-α, IL-6, IL-7, IL-8, IL-10, IL-12, IL-13, IL-17, IL-1 receptor antagonist (IL-1RA), regulated on activation, normal T-cell expressed and secreted (RANTES), vascular endothelial growth factor (VEGF), granulocyte colony-stimulating factor (G-CSF), recombinant human platelet-derived growth factor-BB (PDGF-BB), eotaxin, interferon gamma-induced protein 10 (IP-10), macrophage inflammatory protein (MIP)-1α, MIP-1β, and MCP-1. All testing was performed in duplicate. Results are reported as mean fluorescence intensity and converted to pictogram/mL concentrations using the Bio-Plex Manager version 6.0 software.

### 2.4. Data Analysis

Data were analysed using STATA^®^ version 14. Frequencies, median plus interquartile range (IQR), or mean plus standard deviation were calculated for patient characteristics. Patients were divided into two groups based on whether they had a *Legionella* infection. Categorical variables were analysed between the two groups using the Pearson’s Chi-square test, or Fisher’s exact test if n < 5. Normally distributed continuous variables were compared using the T-test, while non-normal data were compared using the Wilcoxon test. Differences between groups were considered statistically significant if *p* ≤ 0.05.

Systemic immunological factors were analysed to determine whether they differed between the two groups (*Legionella*-positive vs. -negative) and if they correlated with clinical outcome (defined as ICU admission). Cytokines and chemokines are reported as median (IQR) and were compared using a two-sample Wilcoxon rank-sum test.

Next, to determine the systemic cytokine profile associated with *Legionella* infection, a principal component analysis (PCA) was conducted. PCA is a widely employed statistical technique used to discern patterns within data, revealing specific constellations of markers that contribute significantly to variations between groups, such as *Legionella*-infected and uninfected individuals [30]. In this analysis, components were extracted using the principal axis method and rotated using the varimax rotation, a linear transformation facilitating a more straightforward interpretation of the data. The rotated factor pattern that accounted for at least 10% of the total variance was chosen. This factor pattern represents a set of cytokines collectively explaining a substantial portion of the differences between *Legionella*-infected and uninfected individuals. A cytokine was considered to load onto a specific factor component wherever the loading value was the highest (i.e., the cytokine/chemokine had a strong association with that component). Subsequently, the inflammatory markers that loaded onto each rotated component were compared between the *Legionella*-positive and -negative groups using the two-sample Wilcoxon rank-sum test. A Poisson regression was then performed to identify which factors associated with poor clinical outcome (ICU admission) or those associated with *Legionella* infection. Covariates selected for the regression analysis were those with *p* < 0.25 in the univariate analysis, or those deemed biologically relevant based on the literature or prior analyses from our group. Categorical variables were directly integrated into the model, while continuous variables were recoded prior to the analysis. Interaction was evaluated using the Mantel–Haenszel test of homogeneity, and if *p* ≤ 0.05, an interaction term was introduced for the corresponding variables. The model was adjusted for confounders and interaction terms, and results are presented as crude and adjusted relative risk (RR) with 95% CI, alongside the corresponding *p* values.

## 3. Results

Forty-seven patients were included in this study, all of which were diagnosed in hospital with HIV and pneumonia (Table 1; see reference [23] for in-depth details). Individuals consisted predominantly of immunosuppressed males (CD4 cell count < 50 cells/μL, IQR 16-101), with a mean age of 35 years, with the most common in-hospital diagnoses consisting of *Mycobacterium tuberculosis* or *Pneumocystis jirovecii* infection.

Patients were divided into two groups based on our retrospective analysis: (1) *Legionella*-positive individuals with HIV and pneumonia (n = 17); and (2) *Legionella*-negative individuals with HIV and pneumonia (n = 30). Based on patient clinical charts, individuals with *Legionella* infections had higher rates of ICU admissions compared to *Legionella*-negative individuals (n = 9 [53%] vs. n = 7 [23%], respectively; *p* = 0.04) as well as higher mortality rates (n = 9 [53%] vs. n = 6 [20%], respectively; *p* = 0.02).

Of the 19 measured systemic inflammatory markers, the majority were higher in the *Legionella*-positive patients, of which only 2 were significantly different between the patient groups, namely IL-7 and eotaxin (Table 2). Individuals with *Legionella* infection had higher IL-7 plasma levels compared to *Legionella*-negative individuals (0.50 [IQR 0.24–1.91] and 0.24 [IQR 0.24–0.25] pg/mL, respectively, *p* = 0.04). Likewise, plasma eotaxin was also higher among the *Legionella*-infected population (5.27 [IQR 3.58–17.13] vs. 3.08 [IQR 2.06–3.86] pg/mL, *p* < 0.001).

The PCA of plasma immune markers in *Legionella*-positive and -negative patient samples at hospital admission can be seen in Table 3. PCA identified five principal components which had eigenvalues greater than one that were included in the analysis. Together, the five factors accounted for 83.9% of the total variance (22.2%, 20.6%, 20.2%, 10.7% and 10.3% for Factors 1, 2, 3, 4, and 5, respectively). In the rotated factor analysis, six molecules loaded onto component 1 (IL-7, IL-10, IL-12, IL-13, RANTES, and VEGF), four onto component 2 (IL-6, G-CSF, IFN-γ, and TNF-α), four onto component 3 (IL-17, MIP-1α, PDGF-BB, and MIP-1β), three onto component 4 (eotaxin, IP-10, and MCP-1), and two loaded onto the last component (IL-1RA and IL-8).

Of the five component factors, Factor 4 was the only one that was significantly different between the *Legionella* positive and negative groups (*p* = 0.622, *p* = 0.376, *p* > 0.999, *p* = 0.005, and *p* = 0.757, for factors 1, 2, 3, 4, and 5, respectively).

In a binomial logistic regression analysis, higher plasma concentrations of Factor 4 (eotaxin, IP-10 and MCP-1) associated with higher rates of ICU admission. Moreover, individuals with this immune profile had a 5% higher risk of ICU admission, even after controlling for potential confounders such as *Legionella* infection, viral load, CD4 count, and TB coinfection (crude RR 1.021 [CI 1.011–1.031] vs. adjusted RR 1.047 [CI 1.027–1.066]).

## 4. Discussion

Despite being a major cause of respiratory-associated drinking water outbreaks [32], an understanding of host immunity to *Legionella* spp. infections, particularly in HIV-infected individuals, is lacking. To expand our knowledge base, we studied the plasma cytokine response in *Legionella*-infected individuals with HIV-associated pneumonias.

Previous studies looking at cytokines involved in *Legionella* immunity in immune-competent individuals found that *Legionella*-positive individuals had high serum levels of TNF-α, IFN-γ, and IL-12 during infection [13], while in vitro and in vivo studies reported elevated levels of TNF-α, IFN-γ, IL-2, IL-4, IL-6, IL-8, IL-12, IL-18, and MCP-1 [15,18,33]. The present research builds on previously reported work by adding evidence on the systemic involvement of MCP-1, IP-10, and eotaxin. As mononuclear phagocytes are important cells in *Legionella* infection, it is not surprising that chemokines that attract or stimulate the formation of these cell types associated with *Legionella* infection. Indeed, MCP-1 is a key marker regularly involved in monocyte and macrophage recruitment and infiltration [34]. Although it is primarily secreted by phagocytic inflammatory cells, it is also produced by lymphocytes, fibroblasts, and airway endothelial and epithelial cells, following monocyte chemotaxis and oxidative stress [35,36,37,38]. In a study by Matsunaga et al., MCP-1 was found to be upregulated in macrophage cells following stimulation with *Legionella* lipopolysaccharide [33]. This chemokine has also been linked to several pulmonary comorbidities including interstitial lung disease [36], acute lung injury [38], and intracellular infections [35,36,37]. Furthermore, a recent publication by Allam et al. [39] involving 84 hospitalized patients with *Legionella* pneumonia reported that individuals admitted to the ICU with mechanical ventilation had increased levels of plasmatic MCP-1, MIP-1β, IL-6, IL-8, IFN-γ, TNF-α, and IL-17. They also highlighted that upon cell stimulation, *Legionella*-positive patients exhibited elevated release of IL-18 and MCP-1 compared to healthy individuals.

Similarly, a previous study by Lettinga and co-workers which also looked at IP-10 and its association with *Legionella* infection found that IP-10 release following whole blood stimulation with IFN-γ weakly correlated with the severity of *Legionella* pneumonia [40]. IP-10, expressed by Th1 cells, monocytes, fibroblasts, and endothelial cells, plays a crucial role in inflammation by recruiting T lymphocytes and monocytes to the site of infection [41]. It activates T-cells, inducing IFN-γ release and enhancing macrophage activity through a positive feedback loop mechanism [42]. Earlier research has highlighted the essential role of the IP-10 signaling pathway in eradicating intracellular infections [41,43]. During infection, IP-10 orchestrates the adaptive immune response by triggering the mitogen-activated protein kinase and phosphoinositide 3-kinase pathways, thereby affecting chemotaxis, apoptosis, and cell proliferation as well as various cell types (natural killer cells, dendritic cells, and B cells) [43].

In contrast, an association between eotaxin and *Legionella* has not yet been described; however, higher levels have been reported in severe community-acquired pneumonia and *M. tuberculosis* infections [22,44]. Eotaxin upregulation has also been reported in other intracellular infections as well [45,46,47]. Induced by cell stimulation with IFN-γ, eotaxin is produced by endothelial and epithelial cells, eosinophils, monocytes, and macrophages [48]. Once produced, this small protein binds to the CCR3 receptor present on eosinophils, basophils, Th1 and Th2 cells, and airway epithelium [48], initiating a cascade that activates the mitogen-activated protein kinase pathway, increases oxygen radical species production, induces monocytes, recruits eosinophils, basophils, and Th2 lymphocytes, and triggers eosinophil granule release [48,49]. Considering the insights gained in this study and the role that eotaxin, IP-10, and MCP-1 play in the immune response, the evidence suggests that markers of monocyte and macrophage activation and differentiation may play a role in *Legionella* pneumonia pathogenesis and host immunity, warranting further investigation.

An additional intriguing finding is the higher ICU admission rates among *Legionella*-infected individuals, with those having a Factor 4 profile facing an increased risk of being admitted to the ICU. These findings are in agreement with Wolter et al., which showed that *Legionella* patients had case-fatality ratios twice that of individuals who had other severe respiratory illnesses [50]. Moreover, as was seen in our patient population, they found that *Legionella*-positive cases often occurred in coinfected individuals (67%) with HIV, TB, or both HIV and TB.

This work contributes to the existing knowledge surrounding *Legionella* infection and describes for the first time, to the best of our knowledge, systemic cytokine profiles in the context of *Legionella* and HIV coinfections. Another strength of this research lies in the fact that *Legionella* identification and cytokine analysis were performed independently by two researchers who were blinded to the results until all samples were completed. However, a major limitation of this study is that, since it was based on a previously established cohort, we were unable to include individuals with *Legionella* monoinfection (in HIV-infected and HIV-uninfected individuals) in the analysis. Without comparison with these groups, the observations herein cannot definitely state whether the association between Factor 4 and *Legionella*-infected individuals is based on the *Legionella* and HIV coinfection or due to the *Legionella*-induced pneumonia irrespective of HIV. Additionally, due to the retrospective nature of the study, we cannot establish a causal relationship between *Legionella* infection and the Factor 4 profile. Thirdly, as we had a finite volume of BAL samples to which we had access, cytokine analysis could only be performed on patient plasma samples; therefore, we were only able to look at systemic cytokine response which may differ from that seen at the local level. Lastly, our patient population was small and fairly homogenous in that the majority of patients were highly immunosuppressed males with similar coinfections (all with HIV, and most with either tuberculosis or *Pneumocystis jirovecii* pneumonia); therefore, data may not be extrapolated to other patient populations. Other researchers are encouraged to verify our findings as very few groups have studied Legionella coinfection among people living with HIV.

While acknowledging its limitations and exploratory nature, this study provides valuable insights, suggesting that plasmatic markers of monocyte and macrophage activation and differentiation (eotaxin, IP-10 and MCP-1) are associated with *Legionella* infection in individuals coinfected with HIV and *M. tuberculosis* or *P. jirovecii* infection. Furthermore, it indicates that this systemic cytokine pattern is linked to more severe outcomes. Although further work is needed to explore and validate these findings in additional, larger, and prospective cohorts, this study marks an important step towards better understanding cytokines associated with *Legionella* infection in immunocompromised individuals. The insights gained from this work can serve as a foundation for future studies investigating the role that *Legionella* spp. play in inflammation and disease in people living with HIV.

## Figures and Tables

**Table 1:** Demographics and clinical characteristics of all HIV and pneumonia patients with and without *Legionella* infection.

	All Patients (n = 47)	*Legionella*-Negative (n = 30)	*Legionella*-Positive (n = 17)	*p* Value ^3^
**Demographics**				
Age, mean (SD ^2^), years	35.7 (8.8)	36.7 (8.4)	33.9(9.5)	0.279
Male, n (%)	38 (81)	27 (90)	11 (65)	0.054
CD4 cell count (median cells/μL, IQR ^1^)	36.5 (16–101)	25 (12–92.8)	32.5 (16–90.5)	0.957
**Pneumonia (n,%)**				
*Pneumocystis jirovecii*	15 (32)	9 (30)	6 (35)	0.708
*Myco* *bacter* *ium tuberculosis*	19 (40)	9 (30)	10 (59)	0.054
Other	13 (28)	12 (40)	1 (5.9)	0.012
**Outcome**				
ICU admission, n (%)	16 (34)	7 (23)	9 (53)	0.040
Intubation, n (%)	6 (13)	3 (10)	3 (18)	>0.999
Mechanical ventilation, n (%)	4 (8.5)	2 (6.7)	2 (12)	0.613
Death, n (%)	15 (32)	6 (20)	9 (53)	0.020

^1^ IQR: interquartile range; ^2^ SD: standard deviation; ^3^ *p* values comparing *Legionella* positive and negative groups; table adapted from Head et al. [23] and reference [31].

**Table 2:** Plasma inflammatory mediator concentrations for *Legionella*-positive and -negative coinfected individuals at hospital admission.

	*Legionella*-Positive Median (IQR ^1^) pg/mL	*Legionella*-Negative Median (IQR) pg/mL	*p* Value ^2^
IL-1Ra	59.9 (34.41–216.72)	63.72 (30.28–166.87)	0.65
IL-6	11.43 (1.68–69.61)	4.13 (0.94–11.47)	0.22
IL-7	0.50 (0.24–1.91)	0.24 (0.24–0.25)	0.04
IL-8	235.7 (91.54–438.18)	137.71 (27.59–436.19)	0.45
IL-10	1.66 (1.12–2.39)	1.29 (1.09–1.67)	0.28
IL-12	4.45 (3.48–9.31)	4.55 (2.85–6.98)	0.77
IL-13	2.01 (1.45–3.26)	1.43 (0.99–3.81)	0.27
IL-17	4.03 (2.28–5.09)	2.87 (2.20–3.65)	0.43
eotaxin	5.27 (3.58–17.13)	3.08 (2.60–3.86)	<0.001
GCSF	44.27 (11.29–73.41)	20.39 (9.66–48.18)	0.39
IFN-y	1.11 (1.1–8.75)	1.10 (1.10–2.22)	0.18
IP-10	2017.09 (385.88–7902.06)	496.58 (49.56–1618.3)	0.07
MCP-1	133.84 (64.57–464.29)	98.94 (46.7–211.93)	0.12
MIP-1a	3.66 (1.83–5.85)	1.66 (1.14–3.92)	0.07
PDGFBB	2.87 (2.02–6.48)	2.55 (1.6–7.08)	0.50
MIP-1b	52.97 (15.86–82.19)	18.03 (8.46–46.59)	0.14
RANTES	182.35 (80.08–293.91)	125.5 (22.87–269.71)	0.44
TNF-a	2.49 (1.63–3.46)	2.12 (1.43–3.22)	0.46
VEGF	38.08 (23.69–94.31)	59.0 (23.56–129.79)	0.54

^1^ IQR: interquartile range; ^2^ Two-sample Wilcoxon rank-sum (Mann–Whitney) test.

**Table 3:** Principal component analysis of plasma immunological markers in *Legionella*-positive and -negative patient samples at hospital admission.

Rotated Component Matrix					
Variable	Factor1	Factor2	Factor3	Factor4	Factor5
IL-7	0.8669	−0.1346	0.1715	0.1748	0.0641
IL-10	0.7710	0.3706	0.2238	0.2895	0.2582
IL-12	0.9091	0.0897	0.1307	0.1146	0.0667
IL-13	0.6562	0.2333	0.4058	0.0078	−0.1972
RANTES	0.7304	−0.0863	0.3711	0.0116	−0.2910
VEGF	0.6576	0.0445	0.0447	−0.1978	0.6067
IL-6	0.0322	0.9334	−0.0411	0.2984	−0.0054
G-CSF	0.0727	0.9546	0.0545	0.1163	−0.0033
IFN-γ	0.0425	0.8867	0.3215	0.2580	0.0208
TNF-α	−0.0023	0.7050	0.0396	−0.0768	0.3273
IL-17	0.2806	0.4328	0.7831	0.1716	0.0922
MIP-1α	0.1288	0.0262	0.9355	0.0349	0.0392
PDGF-BB	0.6281	0.0282	0.7200	0.0162	−0.1241
MIP-1β	0.2436	0.0387	0.9120	0.1304	0.0418
eotaxin	0.2457	0.3161	0.0072	0.8240	0.2004
IP-10	0.0446	0.4256	0.3826	0.7481	0.0394
MCP-1	0.1647	0.3625	0.1412	0.5934	−0.1809
IL-1RA	0.0171	0.0285	0.0090	0.1310	0.9320
IL-8	−0.0324	0.1452	0.5622	0.1697	0.5624

Note: Molecules that loaded onto each component are shaded. Extraction method: principal component analysis. Rotation method: varimax with Kaiser normalization.

## Data Availability

The data supporting the findings of this study are available within the article as well as in Head et al. [23].
